# Efficacy of probiotics combined with enteral nutrition therapy on intestinal flora, digestive tract symptoms and endogenous environment in patients with gastric cancer undergoing chemotherapy

**DOI:** 10.12669/pjms.40.10.9032

**Published:** 2024-11

**Authors:** Lan Yang, Yuzhu Liang, Riheng Li, Yuan Chen, Xia Zhang

**Affiliations:** 1 Lan Yang, Department of Nutritional, Affiliated Hospital of Hebei University, Baoding 071000, Hebei, China; 2 Yuzhu Liang, Department of Nutritional, Affiliated Hospital of Hebei University, Baoding 071000, Hebei, China; 3 Riheng Li, Department of Stomach Enterochirurgia, Affiliated Hospital of Hebei University, Baoding 071000, Hebei, China; 4 Yuan Chen, Department of Stomach Enterochirurgia, Affiliated Hospital of Hebei University, Baoding 071000, Hebei, China; 5 Xia Zhang, Department of Nutritional, Affiliated Hospital of Hebei University, Baoding 071000, Hebei, China

**Keywords:** Probiotics, Enteral nutrition therapy, Patients with gastric cancer undergoing chemotherapy, Intestinal flora, Endogenous environment

## Abstract

**Objective:**

To investigate the effects of probiotics combined with enteral nutrition therapy on intestinal flora, digestive tract symptoms and endogenous environment in patients with gastric cancer undergoing chemotherapy.

**Methods:**

In this retrospective study, eighty patients with gastric cancer undergoing chemotherapy admitted to Affiliated Hospital of Hebei University from January 2021 to September 2023 were included and divided into two groups by random number table method: the enteral nutrition group and the probiotics combined enteral nutrition group (n=40 each group). The differences in the number of intestinal flora, nutritional indicators, immune function, endotoxin, D-lactic acid levels between the two groups, and the digestive tract symptoms of the two groups before and after treatment were compared and recorded.

**Results:**

The levels of serum total protein (TP), prealbumin (PAB), albumin (ALB) and transferrin (TF) in both groups were higher than those before treatment, and those in the probiotics combined with enteral nutrition group were higher than those in the enteral nutrition group (p< 0.05). The levels of immunoglobulin A (lgA), immunoglobulin M (lgM) and immunoglobulin G (lgG) in the two groups were lower than before treatment, and those in the probiotics combined with the enteral nutrition group were lower than those in the enteral nutrition group (p< 0.05).

**Conclusion:**

Probiotics combined with enteral nutrition results in various benefits in the treatment of patients with gastric cancer undergoing chemotherapy, improving immunity, and reducing gastrointestinal symptoms.

## INTRODUCTION

Gastric cancer, a malignant tumor originating from the gastric mucosal epithelium, occurs in any part of the stomach. It generally has no obvious symptoms in the early stage and is easy to be confused with chronic diseases such as gastritis and gastric ulcer, resulting in a low early diagnosis rate.^1^ Currently, chemotherapy is used as a crucial treatment modality for patients with intermediate and advanced gastric cancer, which has the advantages of controlling disease progression, alleviating clinical symptoms, and improving patients’ quality of life.^2^,^3^ However, during the course of chemotherapy treatment, patients are susceptible to adverse gastrointestinal reactions due to the effects of chemotherapy drugs and loss of appetite, increasing the risk of malnutrition.^4^ Studies have shown^5^ that nutritional therapy can improve the nutritional status of cancer patients undergoing chemotherapy, improve immunity, and maintain intestinal function. Enteral nutrition, as a way to provide nutrients to the body through the gastrointestinal tract, can promote the absorption of nutrients and maintain the structure of the gastrointestinal mucosal barrier. By doing so, it can improve patients’ ability to tolerate chemotherapy. Some researchers believe^6^,^7^ that the adverse reactions caused by long-term chemotherapy may have a close bearing on the disturbance of the intestinal flora of patients. To this end, the supplement of probiotics can correct the imbalance of intestinal flora, increase the resistance of patients, and reduce the toxic side effects of chemotherapy.

Up to now, there are rare reports on the nutritional treatment of patients with gastric cancer undergoing chemotherapy. In this study, we wanted to compare the effects of single enteral nutrition and probiotics combined with enteral nutrition on intestinal flora, digestive tract symptoms and endogenous environment of patients with gastric cancer undergoing chemotherapy, so as to provide a reference for nutritional treatment of patients with gastric cancer undergoing chemotherapy.

## METHODS

This was a retrospective study. Eighty patients with gastric cancer undergoing chemotherapy admitted to Affiliated Hospital of Hebei University from January 2021 to September 2023, and the 80 patients were divided into the enteral nutrition group(n=40) and the probiotics combined enteral nutrition group(n=40) according to different treatment methods. According to the data of each indicator in the pre-survey, the sample size is estimated by 95% confidence interval, and the largest one is the sample size of the study. The sample size required for each group was ≥40 cases on the basis of Fisher exact probability.

### Ethical Approval:

The study was approved by the Institutional Ethics Committee of Affiliated Hospital of Hebei University (No.: HDFYLL-KY-2023-156; date: August 30, 2023), and written informed consent was obtained from all participants.

### Inclusion criteria:

- Patients meeting the clinical diagnostic criteria for gastric cancer^8^;
- Those with complete clinical data;
- Those who persisted in chemotherapy for more than four cycles;
- Those who themselves and their families knew about this study and signed the consent form.

### Exclusion criteria:

- Patients who had a previous history of gastrointestinal and other digestive system surgery;
- Those with severely impaired bowel function or a history of chronic diseases of the digestive tract;
- Those with endocrine abnormalities;
- Those who could not tolerate or were allergic to the enteral nutrition preparations used in this study.

In the enteral nutrition group, there were 22 males and 18 females, aged 48 to 70 years old, with an average age of (57.64 **±** 8.29) years. In the probiotics combined with enteral nutrition group, there were 21 males and 19 females, aged 50 to 72 years old, with an average age of (58.28 **±** 8.34) years. No statistically significant difference was observed in the general information such as gender and age between the two groups, which was comparable (*p>* 0.05).

### Treatment methods:

With reference to the “Guidelines for Nutritional Treatment of Patients with Gastric Cancer”,^9^ treatment plans were developed for both groups. The nutritional status of patients in the two groups was monitored, and the patients were instructed to have a balanced diet and try their best to ensure their energy intake, which ensured that the daily energy intake of patients reached 1500-200 kcal/kg and that of protein was 1.5-2.0g/kg. Patients in the enteral nutrition group were given a whole protein enteral nutrition agent (Milupa GmbH, Germany, National Medical Products Administration No. H20090007), which was dissolved in warm boiled water. Those who could not take it orally were given tube feeding, and a feeding tube was placed in the upper part of the stomach, duodenum or jejunum. The initial dropping speed was set at 25-50 ml/h, and it was gradually increased to 100-125 ml/h according to patients’ tolerance. Patients in the probiotics combined with the enteral treatment group were given bifidobacterium triple viable capsules (Shanghai Xinyi Pharmaceutical Co., Ltd., National Medical Products Administration No. S10970104) on the basis of the enteral nutrition group. Capsules were taken orally, one tablet at a time, tid. Those who could not take it orally were given tube feeding after being fully dissolved in warm boiled water. Both groups were treated for 14 days.

### Observation indicators:

### Number of intestinal flora:

3-5 g of fresh feces were collected from patients before and after treatment and diluted for bacterial culture. Culture plates include E. coli, staphylococcus, enterococcus, etc. After 48 hour of culture, the number and distribution of bacteria on the medium were observed, and CFU/g was used to represent the number of bacteria in 1g of feces.

Detection of nutritional indicators, immune function, endotoxin and D-lactic acid: 3ml of fasting elbow venous blood was collected from patients in the early morning before and after treatment. The serum levels of Total Protein (TP), Prealbumin (PAB), albumin (ALB) and Transferrin (TF) in the two groups were detected by an automatic biochemical analyzer (Boke Biological Industry Co., Ltd., Model: BK-600). Enzyme-linked immunosorbent assay was utilized to detect the levels of Immunoglobulin A (lgA), Immunoglobulin M (lgM), Immunoglobulin G (lgG), endotoxin and D-lactic acid in the two groups.

### Gastrointestinal symptoms:

The incidence of appetite loss, intestinal obstruction, nausea and vomiting, and gastrointestinal discomfort in the two groups after treatment were recorded, and the incidence of gastrointestinal symptoms was calculated. Incidence of gastrointestinal symptoms = [(number of cases of appetite loss + intestinal obstruction + peripheral neuritis + gastrointestinal discomfort)/total number of cases] x 100%.

### Statistical analysis:

All data in this study were statistically analyzed using SPSS22.0 software. Counting data were represented by frequency (rate) and the Chi-square test was used. The significance level α was set to 0.05 and the confidence interval was 95%. Measurement data were expressed as mean ± standard deviation, and t test was used for statistical analysis. *P<* 0.05 was considered a statistically significant difference.

## RESULTS

No statistically significant difference was observed in the intestinal flora between the two groups before treatment (*p>* 0.05). After treatment, the levels of staphylococcus, Escherichia coli and enterococcus in both groups were lower than before treatment, and those in the probiotics combined with enteral nutrition group were lower than those in the enteral nutrition group (*p<* 0.05). Table-I

**Table-I:** Comparison of the number of intestinal flora between the two groups before and after treatment (CFU/g, *χ̅*±*S*).

Group	Number of cases	Escherichia coli		Staphylococcus		Enterococcus	

		Before treatment	After treatment	Before treatment	After treatment	Before treatment	After treatment
Enteral nutrition group	40	8.58±0.96	7.78±0.75*	4.60±0.59	4.02±0.39*	9.58±1.02	9.14±0.83*
Probiotics combined with enteral nutrition group	40	8.64±0.97	8.22±0.71*	4.72±0.54	4.26±0.35*	9.62±1.01	8.65±0.77*
*t*		0.278	2.695	0.949	2.897	0.176	2.737
*p*		0.782	0.009	0.346	0.005	0.861	0.008

***Note:***

* p< 0.05 compared with before treatment in the same group.

No statistically significant difference was observed in the nutritional indicators between the two groups before treatment (*p>* 0.05). The levels of TP, PAB, ALB and TF in both groups were higher than those before treatment, and those in the probiotics combined with enteral nutrition group were higher than those in the enteral nutrition group (*p<* 0.05). Table-II

**Table-II:** Comparison of nutritional indexes between the two groups before and after treatment (*χ̅*±*S*).

Group	Number of cases	TP (g/L)		PAB (ng/L)	

		Before treatment	After treatment	Before treatment	After treatment
Enteral nutrition group	40	69.94±3.03	73.57±1.72*	240.68±20.58	284.24±20.47*
Probiotics combined with enteral nutrition group	40	69.87±3.01	75.49±1.98*	243.41±20.74	317.59±20.50*
*t* value		0.104	4.630	0.591	7.281
*p* value		0.918	0.000	0.556	0.000

***Group***	***Number of cases***	***ALB (g/L)***		***TF (g/L)***	

		***Before treatment***	***After treatment***	***Before treatment***	***After treatment***

Enteral nutrition group	40	37.59±2.94	42.18±2.47*	2.75±0.64	3.34±0.52*
Probiotics combined with enteral nutrition group	40	37.63±2.88	45.23±2.58*	2.78±0.61	3.97±0.54*
*t* value		0.046	5.401	0.215	5.315
*p* value		0.963	0.000	0.831	0.003

***Note:***

* p< 0.05 compared with before treatment in the same group.

No statistically significant difference was observed in the immune function indicators between the two groups before treatment (*p>* 0.05). The levels of IgA, IgM and IgGin the two groups were lower than before treatment, and those in the probiotics combined with enteral nutrition group were lower than those in the enteral nutrition group (*p<* 0.05). Table-III

**Table-III:** Comparison of immune function between the two groups before and after treatment (g/L,χ̅±*S*).

Group	Number of cases	lgA		lgM		lgG	

		Before treatment	After treatment	Before treatment	After treatment	Before treatment	After treatment
Enteral nutrition group	40	2.29±0.54	2.01±0.30*	1.19±0.29	0.89±0.21*	10.52±2.15	9.47±1.22
Probiotics combined with enteral nutrition group	40	2.27±0.55	1.75±0.34*	1.21±0.28	0.72±0.22*	10.34±2.20	8.69±1.37
*t* value		0.164	3.627	0.314	3.535	0.370	2.689
*p* value		0.870	0.001	0.755	0.001	0.712	0.009

***Note:***

* p< 0.05 compared with before treatment in the same group.

No statistically significant difference was observed in the levels of endotoxin and D-lactic acid between the two groups before treatment (*p>* 0.05). The levels of endotoxin and D-lactic acid in both groups were lower than before treatment, and that in the probiotics combined with enteral nutrition group was lower than that in the enteral nutrition group (P<0.05). Table-IV. The incidence of gastrointestinal symptoms in the enteral nutrition group was 22.50% (9/40), which was lower than 5.00% (2/40) in the probiotics combined with the enteral nutrition group (*p<* 0.05). Table-V

**Table-IV:** Comparison of the levels of endotoxin and D-lactic acid between the two groups before and after treatment (*χ̅*±*S*).

Group	Number of cases	Endotoxin (EU/mL)		D-lactic acid (mmol/L)	

		Before treatment	After treatment	Before treatment	After treatment
Enteral nutrition group	40	0.78±0.21	0.52±0.14	1.87±0.69	1.12±0.19
Probiotics combined with enteral nutrition group	40	0.77±0.20	0.41±0.11	1.89±0.71	0.86±0.18
*t* value		0.218	3.907	0.128	6.283
*P* value		0.828	0.000	0.899	0.000

***Note:*** *p< 0.05 compared with before treatment in the same group.

**Table-V:** Comparison of occurrence of gastrointestinal symptoms between the two groups [*n* (%)].

Group	Number of cases	Appetite loss	Intestinal obstruction	Nausea and vomiting	Gastrointestinal discomfort	Incidence
Enteral nutrition group	40	3 (7.50)	3 (7.50)	1 (2.50)	2 (5.00)	9 (22.50)
Probiotics combined with enteral nutrition group	40	1 (2.50)	0 (0.00)	0 (0.00)	1 (2.50)	2 (5.00)
*x^2^* value						5.165
*p* value						0.023

## DISCUSSION

It was shown in this study that the levels of staphylococcus, *Escherichia coli* and enterococcus in the two groups after treatment were lower than those before treatment, and those in the probiotics combined with enteral nutrition group were lower than those in the enteral nutrition group. The results of this study are consistent with those of Li et al.,^10^ indicating that the use of probiotics combined with enteral nutrition can improve the balance of intestinal flora in patients with gastric cancer undergoing chemotherapy. Some studies have shown^11^ that the addition of probiotics on the basis of enteral nutrition is conducive to the smooth development of enteral nutrition treatment for patients, and helps to increase their tolerance to chemotherapy and improve their nutritional status. The levels of TP, PAB, ALB and TF in the two groups after treatment were higher than those before treatment, and the probiotics combined with the enteral nutrition group were higher than those in the enteral nutrition group, with a statistically significant difference. The results of this study are consistent with those of the above studies, suggesting that probiotics combined with enteral nutrition can better improve the nutritional status of patients with gastric cancer undergoing chemotherapy. The reason may be that while maintaining the balance of intestinal flora, probiotics can also improve gastrointestinal blood supply, boost the recovery of absorption function, and promote the absorption and utilization of intestinal nutrients.^12^

Nutritional therapy refers to a treatment method that supplements or provides nutrients necessary for maintaining the human body through enteral and parenteral nutrition when the patient’s dietary intake is insufficient or unable to be taken in. It is mainly divided into enteral nutrition and parenteral nutrition.^13^ Specifically, parenteral nutrition supplies nutrients through the intravenous route, which allows patients to maintain nutrition and promote wound healing when they are unable to eat normally. However, long-term use of parenteral nutrition can damage patients’ intestinal mucosa and lead to poor prognosis.^14^ In contrast, enteral nutrition is a form of nutritional support that provides metabolically needed nutrients and various other nutrients through the gastrointestinal tract. It is mainly divided into two methods: oral administration and transcatheter infusion. The nutrients provided by enteral nutrition therapy are absorbed and utilized directly through the intestine, which is convenient for administration and more consistent with physiological characteristics. It helps maintain the integrity of the intestinal mucosal structure and barrier function. Therefore, enteral nutrition is the preferred treatment when providing nutritional therapy.^15^ Some scholars believe^16^ that the intestinal flora of patients with gastric cancer is in an unbalanced state, and the progression of the disease may be related to the changes in intestinal flora. Therefore, the regulation of the intestinal flora of patients can improve the clinical symptoms of patients. Studies have shown^17^ that supplementation of probiotics can increase the appetite of patients, maintain the proportion of normal bacteria in the digestive tract, and promote the absorption of gastrointestinal nutrients.

In addition to its role in digestion and absorption, the gastrointestinal tract is also an important immune organ in the body. When patients with gastric cancer undergo chemotherapy, the body may enter a state of stress and cause immune function suppression.^18^ It was shown in this study that the levels of IgA, IgM and IgG of the two groups after treatment were lower than those before treatment, and those in the probiotics combined with enteral nutrition group were lower than those in the enteral nutrition group. This suggests that probiotics combined with enteral nutrition can better regulate the immune function of patients. The reason may be that probiotics can induce non-specific immunity and specific immunity in the body and promote the formation of antibodies. In addition, probiotics can also prevent the synthesis of inflammatory cytokines and enhance the role of natural killer cells, thus improving the body’s immunity.^19^ Endotoxin and D-lactic acid, as metabolic end products of intestinal bacteria, reflect intestinal permeability. Patients with gastric cancer undergoing chemotherapy are prone to intestinal endotoxemia due to decreased intestinal mucosal barrier function, thus stimulating the release of endotoxin and D-lactic acid in the body^20^.

It was shown in this study that the levels of endotoxin and D-lactic acid in both groups after treatment were lower than before treatment, and those in the probiotics combined with enteral nutrition group were lower than those in enteral nutrition group. This suggests that probiotics combined with enteral nutrition can better help restore intestinal mucosal cell function. This study also showed that the incidence of gastrointestinal symptoms in the probiotics combined with enteral nutrition group was lower than that in the enteral nutrition group, indicating that probiotics combined with enteral nutrition could alleviate gastrointestinal symptoms in patients with gastric cancer undergoing chemotherapy.

### Limitations:

It includes small sample size, small course of treatment, and short follow-up time. It still needs further clinical research to observe the long-term clinical effect of Probiotics combined with enteral nutrition therapy for gastric cancer chemotherapy patients, so as to apply a better scheme to patients in need.

## CONCLUSIONS

Probiotics combined with enteral nutrition boasts various benefits in the treatment of patients with gastric cancer undergoing chemotherapy, such as regulating the balance of intestinal flora, ameliorating patients’ nutritional status and endotoxin and D-lactic acid levels, improving immunity, and reducing gastrointestinal symptoms.

### Authors’ Contributions:

**LY and XZ** carried out the studies, data collection, drafted the manuscript, **YL** performed the statistical analysis and participated in its design. Critical Review. **RL and YC** performed the statistical analysis and participated in its design; All authors read and approved the final manuscript and are responsible and accountable for the accuracy or integrity of the work.
